# Apoptosis in ischemic heart disease

**DOI:** 10.1186/s12967-017-1191-y

**Published:** 2017-05-01

**Authors:** Elena Teringova, Petr Tousek

**Affiliations:** Cardiocenter, Department of Cardiology, 3rd Faculty of Medicine, Charles University and University Hospital Kralovske Vinohrady, Srobarova 50, 100 34 Prague 10, Czech Republic

**Keywords:** Apoptosis, Ischemic heart disease, Outcome, Heart failure

## Abstract

Apoptosis plays an important role in the myocardial loss after acute myocardial infarction and participates in the process of subsequent left ventricular remodeling and development of symptomatic heart failure. Finding a sensitive apoptotic marker that would help in prognostic stratification of patients after acute myocardial infarction and offer new therapeutic strategies is thus of a great importance. Several studies suggest that tumor necrosis factor-related apoptosis inducing ligand (TRAIL) represents a very promising marker of prognosis in patients with acute myocardial infarction. This review article provides an overview of current knowledge on the role of apoptosis in ischemic heart disease and highlights potentially beneficial apoptotic markers in clinical practice.

## Background

Ischemic heart disease (IHD) is a leading cause of death worldwide. Acute myocardial infarction (AMI) is the most common first manifestation of IHD and represents a major cause of morbidity and mortality. In the past decades, treatment of patients with AMI has significantly improved. However, more patients subsequently suffer from left ventricular (LV) dysfunction and heart failure [1]. Although necrosis was thought to be the sole cause of death in myocardial infarction for a long time, recent studies provide growing evidence that apoptosis plays an important role in the process of myocyte loss after AMI, as well as in the process of LV remodeling and development of heart failure [2]. Recognizing a sensitive apoptotic marker that would help define high-risk patients after AMI and offer new therapeutic strategies is thus of a great importance. Inhibition of apoptosis through anti-apoptotic therapy could represent a new way forward to improve prognosis of these patients. The aim of this article is to discuss the role of known apoptotic markers in IHD and highlight their potential benefit in clinical practice.

## Main text

### Role of apoptosis in ischemic heart disease

Apoptosis, a form of programmed cell death, represents a highly regulated and energy-requiring process by which activation of specific signaling cascades leads to a cell death [3]. Apoptosis plays an important role in various physiological processes including embryogenesis, normal tissue homeostasis and aging [4–6]. However, excessive or insufficient apoptosis results in many diseases, including cancer, some infectious, autoimmune and neurological diseases (e.g. Parkinson’s disease, Alzheimer’s disease, Huntington’s disease) [7–13].

Several recent studies have also demonstrated an important role of apoptosis in ischemic heart disease. Apoptosis significantly contributes to myocyte cell death in AMI and occurs predominantly in the peri-infarcted region [14, 15]. High grade of apoptosis is present also at the subacute phase of MI [16] and correlates with parameters of progressive LV remodeling [2, 17]. Moreover, patients who developed symptomatic heart failure shortly after AMI were associated with significantly increased apoptotic rates [2]. Thus apoptosis is shown to play an important role in determining infarct size, extent of LV remodeling and development of early symptomatic heart failure after AMI.

### Apoptotic markers in ischemic heart disease

Apoptosis can be initiated through two main pathways: the extrinsic or death receptor pathway and the intrinsic or mitochondrial pathway. Both pathways converge on the same terminal pathway. Markers of apoptosis will be introduced thereinafter according to the pathway they are involved in.

### Apoptotic markers of death receptor pathway

**Fig. 1:**
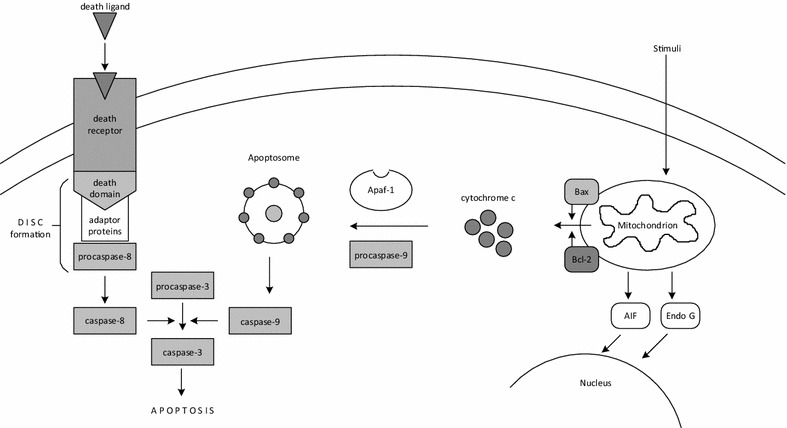
Schematic diagram of apoptotic signaling pathway. Apoptosis can be induced by the extrinsic or death receptor pathway and the intrinsic or mitochondrial pathway. The extrinsic pathway is initiated after an apoptotic ligand (e.g. FasL, TNF-α, TRAIL) binds to its death receptor. Subsequently, death-inducing signaling complex is formed resulting in caspase-8 activation. Caspase-8 activates effector caspases and triggers the terminal phase of the apoptotic cascade. The intrinsic pathway is initiated through wide range of none-receptor mediated stimuli (e.g. deprivation of growth factors, hypoxia, oxidative stress), resulting in changes of the mitochondrial membrane permeability. After its release from mitochondria, cytochrome c together with Apaf-1 and procaspases-9 forms an apoptosome, resulting in caspase-9 activation. Caspase-9 then activates effector caspases, such as caspase-3. AIF and EndoG are also released from mitochondria and translocate to cell nucleus where they cause DNA fragmentation, independently of caspase activation. Both extrinsic and intrinsic pathways converge on the same terminal pathway

Death receptor pathway is significantly involved in inducing myocardial apoptosis in IHD. The mechanism of death receptor pathway has been extensively reviewed in the literature. Briefly, after an apoptotic ligand binds to its death receptor, death-inducing signaling complex is formed, resulting in caspase-8 activation [18]. Caspase-8 activates effector caspases 3 and 7 and thus triggers the terminal phase of the apoptotic cascade (Fig. 1).

### Fas

In experimental studies, apoptosis-stimulating fragment (Fas) is described as a critical mediator of cardiomyocyte apoptosis during ischemia/reperfusion (I/R) injury [19]. However, levels of soluble Fas receptor (sFas) and Fas ligand (FasL) measured in patients with different forms of IHD provided rather controversial results. Levels of sFas were significantly higher in patients with acute MI compared to healthy controls or patients with chronic ischemic heart disease. However, sFas levels failed to correlate with the infarct size. Moreover, no difference was found in serum levels of Fas ligand among patients with acute MI, chronic forms of IHD or healthy controls [20].

In the study by Fertin et al. levels of FasL were measured in patients 1 month after MI and several echocardiographic studies were performed up to 1-year post MI to evaluate LV volumes. LV remodeling was documented by a significant increase of LV volumes; however, changes in LV volumes were not associated with FasL levels [21].

Similar findings were documented by Nilsson et al. who measured levels of both sFas and sFasL in patients with ST-elevation myocardial infarction (STEMI) prior to percutaneous coronary intervention (PCI) and 24 h after the procedure. Cardiac MRI was used to evaluate infarct size and LV dysfunction 5 days and 4 months after STEMI. No correlation was found between sFas or FasL and infarct size or LV dysfunction [22].

In line with this, serum levels of sFas measured in patients with acute coronary syndromes were not associated with patient prognosis during 6-month follow-up [23].

In conclusion, sFas levels are significantly higher in patients with acute myocardial infarction but failed to correlate with the infarct size, LV dysfunction, or prognosis. Thus the role of Fas-mediated apoptosis in IHD is yet unclear.

However, sFas levels seem to be helpful as a prognostic marker in patients with various forms of heart failure. Prognostic value of sFas was demonstrated in patients with dilated cardiomyopathy, decompensated heart failure, as well as in patients with compensated heart failure [24–26]. Higher sFas concentrations were associated with higher risk of mortality or hospitalization for heart failure.

### TNF-α

Experimental studies also demonstrate an important role of tumor necrosis factor alpha (TNF-α) in mediating apoptosis of cardiomyocytes during I/R. TNF-α is a proinflammatory cytokine with various biological functions. After binding to its receptor, TNF-α can induce apoptosis in cardiomyocytes, as shown in an in vitro rat study [27]. TNF-α shows dual effect in the heart depending on its concentration and on the type of its receptor. Low dose of TNF-α improves myocardial function while high dose of TNF-α increases myocardial injury following ischemia and reperfusion [28]. In an in vivo murine model of myocardial infarction, TNF-α showed its cardiotoxic effect through receptor TNFR1 and cardioprotective effect through TNFR2. Mice lacking TNFR2 demonstrated worse post-IM survival, more severe ventricular dysfunction, and exacerbated myocyte hypertrophy and interstitial fibrosis in non-infarct myocardium—compared to TNFR1-knockout mice [29].

Studies in heart failure patients demonstrate that high levels of TNF-α are expressed within failing human myocardium, suggesting its role in the progression of heart failure [30, 31]. However, anti-TNF-α clinical trials using TNF-α antagonist or TNF-α antibody showed no benefit to patients with heart failure [32, 33]. These disappointing results support the presumption of ambivalent effects of TNF-α in the cardiovascular system.

Levels of TNF-α and its receptors are elevated also in patients with acute myocardial infarction and can predict infarct size, LV dysfunction and prognosis [22, 34, 35]. A study by Kehmeier et al. showed that TNF-α levels measured in STEMI patients after PCI can predict infarct size [34]. Nilsson et al. measured soluble TNF-receptors in STEMI patients prior to and 24 h after PCI and showed that concentrations of TNFR1 and TNFR2 are associated with infarct size and LV dysfunction [22].

TNFR1 has proven to have a prognostic value in patients after acute myocardial infarction. Valgimigli et al. show that plasma levels of soluble TNFR1 are a predictor of mortality and new onset of heart failure in patients with AMI [36]. Ueland et al. demonstrate that levels of sTNFR1 can predict all-cause mortality and cardiovascular death in patients who developed heart failure after AMI [37].

Other studies suggest that also TNF-α has a prognostic value in patients with AMI [38–40]. However, a clinical trial with TNF-α antagonist in patients with acute myocardial infarction provided no evidence of immediate beneficial effect for these patients [41].

These findings suggest that involvement of TNF-α in ischemic heart disease is very complex and includes a wide range of biological processes—both harmful and beneficial. Further research is needed to clarify the exact molecular mechanism of TNF-α in IHD and find possible ways to inhibit only the negative TNF-α effects.

Since TNF-α is involved in IHD pathological pathway, TNF-α gene polymorphisms and their association with the risk of myocardial infarction and coronary artery disease (CAD) have been extensively studied. A meta-analysis investigating relationship between TNF-α gene polymorphisms and CAD risk, however, showed no association [42].

### TRAIL

TNF-related apoptosis stimulating ligand (TRAIL) is a member of TNF superfamily that can induce apoptosis. After binding to its receptors TRAIL-R1 and TRAIL-R2, TRAIL initiates intracellular signaling cascade resulting in the apoptotic cell death [43, 44]. In an I/R model in isolated rat and mouse hearts, TRAIL was released from the postischemic hearts early after the onset of reperfusion [45]. However, the exact molecular mechanism of TRAIL has not yet been completely understood. Some experimental data suggest that TRAIL-R1 and TRAIL-R2 can also mediate cell type-dependent prosurvival and proliferation signals [46]. In experimental studies, administration of soluble recombinant TRAIL showed protective activity. In a diabetic mouse model, direct administration of TRAIL reduced development of cardiomyopathy [47]. Another similar study demonstrated that systemic TRAIL delivery showed anti-atherosclerotic activity in diabetic mice [48].

Several clinical studies report that levels of TRAIL are decreased in patients with acute myocardial infarction [23, 49, 50]. Moreover, TRAIL is reported to be a potential marker of severity of coronary artery disease and predictor of prognosis in patients after acute myocardial infarction. Mori et al. measured serum TRAIL levels in patients undergoing coronary angiography. TRAIL levels were significantly lower in patients with coronary artery disease compared to those without. Moreover, TRAIL levels were inversely associated with the severity of CAD, suggesting potential use of TRAIL as a marker of CAD severity [49].

Secchierro et al. measured TRAIL in patients with acute myocardial infarction in serial serum samples during hospitalization and in a 12 month-follow-up. Serum levels of TRAIL were significantly decreased in AMI patients at baseline (compared to healthy controls) and low TRAIL levels at patient discharge were associated with increased incidence of cardiac death and heart failure at the 12-month follow-up, even after adjustment for demographic and clinical risk parameters. Thus low TRAIL levels represent a potential predictor of cardiovascular events following acute myocardial infarction [50].

A study by Osmancik et al. provided similar findings. TRAIL levels were measured in acute coronary syndrome patients, who were then followed for 6 months. Low serum TRAIL concentrations were the strongest significant and independent predictor of the composite endpoint of death and hospitalization for heart failure [23].

Low TRAIL levels can predict worse prognosis also in patients with chronic heart failure and in older patients with cardiovascular disease [25, 51].

In conclusion, serum TRAIL levels seem to represent an important predictor of prognosis in patients with acute myocardial infarction. Low TRAIL levels are associated with worse prognosis of AMI patients while higher TRAIL levels seem to be protective. It is unclear whether decreased TRAIL levels reflect reduced production or increased consumption. Metalloproteinase 2, which level is elevated in patients with acute coronary syndromes, can cleave TRAIL, as shown in an in vitro study [52]. This could be a potential explanation of decreased TRAIL level in patients with acute myocardial infarction. Better understanding of the exact molecular mechanism of TRAIL might provide a new target for therapeutic intervention in patients with acute myocardial infarction.

### Apoptotic markers of mitochondrial pathway

The intracellular signaling cascade of mitochondrial pathway is shown in Fig. 1. Activation of mitochondrial pathway and release of pro-apoptotic proteins from mitochondria into the cytosol is controlled and regulated by Bcl-2 family of proteins [53].

### Bcl-2

Experimental studies show that cardiac specific overexpression of Bcl-2, an inhibitor of apoptosis, significantly reduces infarct size after I/R injury. This reduction of I/R injury correlates with the reduction of cardiomyocyte apoptosis [54, 55]. Expression of Bcl-2 was studied also in the hearts of patients who died of MI [56]. However, no clinical study was performed to examine level of Bcl-2 in serum of AIM patients. In cancer patients, evaluation of Bcl-2 serum levels and its tissue expression showed correlation bordering on statistical significance [57].

### Apoptotic markers of terminal pathway

Terminal apoptotic pathway is common for both extrinsic and intrinsic pathway. Activation of execution caspases results in degradation of cellular constituents. This is followed by fragmentation into apoptotic bodies that are quickly removed by phagocytes [58].

### Caspase-3

The most important caspase of the terminal apoptotic pathway is caspase-3. Overexpression of cardiac specific caspase-3 in transgenic mice showed increased infarct size and pronounced susceptibility to die after I/R injury [59]. Vice versa, downregulation of caspase-3 decreased the infarct size, lowered the apoptotic index of myocytes and improved the heart function in an experimental model with myocardial infarction [60].

The cleaved caspase-3 p17 peptide was shown to escape from apoptotic cancer cells and was detectable in extracellular medium after induction of apoptosis, suggesting the concept that p17 peptide can escape also into circulation from apoptotic cells in patients. This presumption was used in a clinical study with 27 STEMI patients undergoing PCI [61]. p17 peptide was measured within initial 24 h and 3 months after STEMI (88 ± 29 days). Compared to healthy subjects, peak p17 levels were nearly fourfold higher in the acute phase of AMI and stayed significantly higher also in the late post-STEMI samples. However, the p17 peptide kinetics and its correlation with the myocyte apoptosis after myocardial infarction have not yet been sufficiently examined.

### Inhibition of apoptosis

Since apoptosis is significantly involved in myocardial injury after AMI, inhibition of apoptosis represents an appealing target for a therapeutic intervention.

#### TNF-α inhibition therapy

TNF-α inhibition therapy was examined in large clinical trials with heart failure patients, using TNF-α antagonist infliximab or TNF-α antibody etanercept. Anti-TNF-α therapy, however, provided no evidence of clinical benefit to heart failure patients.

Two trials RECOVER and RENAISSANCE evaluated a TNF-α antagonist etanercept in 2000 patients with chronic heart failure. Both trials were terminated prematurely because etanercept failed to show clinically relevant benefit on the patient clinical status, the rate of death or hospitalization due to chronic heart failure [32].

ATTACH trial evaluated a TNF-α antibody infliximab in 150 patients with moderate-to-severe heart failure. Results showed that short-term inhibition of TNF-α with infliximab did not improve and high dose adversely affected the clinical condition of examined patients [33].

In patients with acute myocardial infarction, TNF-α antagonist etanercept was used in one trial with 26 patients. Etanercept reduced systemic inflammation markers but increased platelet activation. TNF-α antagonism thus provided no evidence of immediate benefit for AMI patients [41]. Effect of anti-TNF-α therapy on diverse outcomes in AMI patients was not evaluated.

These findings suggest that involvement of TNF-α in the pathogenesis of ischemic heart disease and development of heart failure includes various biological processes and simple inhibition of all TNF-α effects does not provide clinical benefit to cardiac patients.

On the other hand, TNF-α inhibition therapy brought a revolution in the treatment of several autoimmune diseases, such as rheumatoid arthritis, psoriasis and non-specific inflammatory bowel diseases. Clinical trials indicate that anti-TNF-α therapy in patients with psoriasis is associated with significantly decreased risk of myocardial infarction, compared to treatment with topical agents [62, 63]. The risk of myocardial infarction is markedly reduced also in patients with rheumatoid arthritis who respond to anti-TNF-α therapy [64, 65].

#### TRAIL-targeted therapy

No clinical trials have been done using TRAIL as a therapeutic target yet.

## Conclusion

Apoptotic cell death represents a significant contributor to myocardial damage in patients with acute myocardial infarction and participates in the process of subsequent LV remodeling and development of heart failure. Finding a sensitive marker of apoptosis that would help predict prognosis of AMI patients is of a great importance. Among intracellular apoptotic mediators, the only one evaluated in serum of patients with AMI is fragment of caspase-3 p17 peptide. Serum levels of p17 peptide brought some hopeful results in one study with STEMI patients; however, further research is needed to prove p17 peptide as a substantial marker of apoptosis. Among extracellular markers of apoptosis, several soluble forms of receptors and their ligands were evaluated in clinical studies with AMI patients. The most promising apoptotic marker seems to be TRAIL. Serum TRAIL levels represent an important predictor of prognosis in AMI patients. Low TRAIL levels are associated with worse prognosis and high TRAIL levels seem to be beneficial. So far, it is unclear whether decreased TRAIL levels represent a reduced production or an increased consumption. Deeper understanding of the exact molecular mechanism of TRAIL may offer a new target for therapeutic intervention in patients with acute myocardial infarction.

## Abbreviations

AMI: acute myocardial infarction
CAD: coronary artery disease
Fas: apoptosis stimulating fragment
FasL: apoptosis stimulating fragment ligand
I/R: ischemia/reperfusion
IHD: ischemic heart disease
LV: left ventricular
MI: myocardial infarction
PCI: percutaneous coronary intervention
sFas: soluble apoptosis stimulating fragment
STEMI: ST-elevation myocardial infarction
TNF-α: tumor necrosis factor alpha
TNFR1: tumor necrosis factor receptor 1
TNFR2: tumor necrosis factor receptor 2
TRAIL: TNF-related apoptosis stimulating ligand
TRAIL-R1: TNF-related apoptosis stimulating ligand-receptor 1
TRAIL-R2: TNF-related apoptosis stimulating ligand-receptor 2
